# Metal-backed or all-poly tibial components: which are better for medial unicompartmental knee arthroplasty? A propensity-score-matching retrospective study at the 5-year follow-up

**DOI:** 10.1186/s10195-024-00765-3

**Published:** 2024-05-04

**Authors:** Gianluca Piovan, Luca De Berardinis, Daniele Screpis, Marco Senarighi, Lorenzo Povegliano, Simone Natali, Antonio Pompilio Gigante, Claudio Zorzi

**Affiliations:** 1 Department of Orthopaedics, IRCCS Ospedale Sacro Cuore Don Calabria, Negrar Di Valpolicella, Italy; 2 Clinical Orthopaedics, Department of Clinical and Molecular Science, School of Medicine, Università Politecnica Delle Marche, Via Tronto, 10/a, 60126 Ancona, AN Italy

**Keywords:** Unicompartmental knee arthroplasty, All-poly tibial component, Metal-backed tibial component, Propensity score matching, Knee pain, Functional outcomes, Satisfaction, Gait, Postural balance, Inertial measurement unit

## Abstract

**Background:**

This retrospective medium-term follow-up study compares the outcomes of medial fixed-bearing unicompartmental knee arthroplasty (mUKA) using a cemented metal-backed (MB) or an all-polyethylene (AP) tibial component.

**Materials and methods:**

The database of our institution was mined for primary mUKA patients implanted with an MB or an AP tibial component (the MB-UKA and AP-UKA groups, respectively) from 2015 to 2018. We compared patient demographics, patient-reported outcome measures (PROMs), and motion analysis data obtained with the Riablo™ system (CoRehab, Trento, Italy). We conducted propensity-score-matching (PSM) analysis (1:1) using multiple variables.

**Results:**

PSM analysis yielded 77 pairs of MB-UKA and AP-UKA patients. At 5 years, the physical component summary (PCS) score was 52.4 ± 8.3 in MB-UKA and 48.2 ± 8.3 in AP-UKA patients (*p* < 0.001). The Forgotten Joint Score (FJS-12) was 82.9 ± 18.8 in MB-UKAs and 73.4 ± 22.5 in AP-UKAs (*p* = 0.015). Tibial pain was reported by 7.8% of the MB-UKA and 35.1% of the AP-UKA patients (*p* < 0.001). Static postural sway was, respectively, 3.9 ± 2.1 cm and 5.4 ± 2.3 (*p* = 0.0002), and gait symmetry was, respectively, 92.7% ± 3.7 cm and 90.4% ± 5.4 cm (*p* = 0.006). Patient satisfaction was 9.2 ± 0.8 in the MB-UKA and 8.3 ± 2.0 in the AP-UKA group (*p* < 0.003).

**Conclusions:**

MB-UKA patients experienced significantly better 5-year static sway and gait symmetry outcomes than AP-UKA patients. Although the PROMs of the two groups overlapped, MB-UKA patients had a lower incidence of tibial pain, better FJS-12 and PCS scores, and were more satisfied.

## Introduction

Osteoarthritis of the knee (KOA) is highly prevalent on a global scale and is the primary cause of musculoskeletal disability. It limits physical and work activity, affects quality of life, and involves elevated healthcare expenses [1–3]. When conservative approaches prove ineffective, surgical intervention, in the form of partial or total knee replacement, becomes necessary. Both procedures are common in developed countries, and their prevalence is expected to rise substantially in the future [2, 4]. Unicompartmental knee arthroplasty (UKA) has garnered increasing popularity in recent times, as multiple studies have indicated that it is less invasive, involves a shorter duration of surgery, provides a wider range of motion, offers greater pain relief, enables a quicker return to daily activities and sports, and is less expensive than total knee arthroplasty (TKA) [5–11].

In about 90% of cases, UKA is performed on the medial tibiofemoral joint [12, 13]. Among the decisions to be made in the surgical approach to UKA are the implant fixation method (cemented or uncemented), the design (mobile or fixed bearing), and the placement (conventional or robotic).

In fixed-bearing UKA, the tibial component is usually either all polyethylene (AP) or metal backed (MB). Whereas the former component is thicker, ensuring preservation of the bone stock for eventual surgical revision, and is associated with a lower implant cost [14, 15], the latter provides greater flexibility during surgery and allows for separate bearing replacement [16]. However, an MB component has potential issues with wear on the backside of the interface [17], greater bone loss in case of implant failure [18], and a higher cost [19]. Two studies have found that AP components provide slightly better functional outcomes than MB components [16, 20], whereas biomechanical studies have demonstrated greater stress and strain on the proximal tibial cancellous bone in patients with AP components [19, 21, 22]. Elevated tibial bone strain and microdamage have been suggested as factors that contribute to unexplained pain and suboptimal outcomes [23, 24]. Whereas one study has described better functional outcomes with an MB than with an AP tibial component at medium-term follow-up [25], other studies, including a meta-analysis, have found no discernible differences in functional outcomes between them [14, 24, 26–28].

In recent years, instrumented motion analysis has increasingly been used to gain an objective and quantitative description of an individual’s motor functions and abilities [29–31]. This method plays a key role in the evaluation of pathological conditions, compensatory motor strategies, and the monitoring of improvement during rehabilitation. It also offers a more sensitive and objective means of assessment compared to the ordinal scoring used in “semiquantitative” clinical scales [32]. Critically, instrumented motion analysis allows the kinematic and kinetic parameters of human movements to be determined and musculoskeletal functions to be assessed in a quantitative manner. Consequently, it has found numerous applications in clinical assessments, rehabilitation, sports, and even diagnostics [33].

Since the turn of the new millennium, technological advances in motion measurement techniques have facilitated the assessment of body segment kinematics through wearable inertial sensors that are miniaturized inertial measurement units (IMUs), which quantify three-dimensional linear acceleration and angular velocity with respect to the axes of a sensor-embedded frame of reference [34].

In the coming years, these devices are expected to see ever-wider clinical utilization through integration into various devices designed specifically for clinical interventions (particularly rehabilitation), thus extending beyond research and motor function assessment. Several companies have incorporated IMUs into video-game-based rehabilitation systems such as the Riablo™ system (CoRehab, Trento, Italy) [35]. This trend reflects the growing awareness of the potential of wearable inertial devices to enhance clinical interventions and rehabilitation.

The aim of this study was to determine whether there is a difference in medium-term functional outcome scores, health-related quality of life (HRQL) scores, and static postural sway, mobility, and gait symmetry between medial UKA (mUKA) patients implanted with an MB or an AP tibial component.

## Materials and methods

### Patient selection

Following approval by the institutional review board, the database of the Orthopedic Department of IRCCS Sacro Cuore-Don Calabria Hospital (Negrar di Valpolicella, Italy) was mined for all primary mUKAs performed from 2015 to 2018. There were 653 such procedures, 342 involving an MB and 311 involving an AP tibial component.

The data collected included demographics, body mass index (BMI), medical history, American Society of Anesthesiologists (ASA) class, operative time, length of hospital stay, any revision surgeries or complications, clinical outcomes, and patient satisfaction.

### Indications of surgery

All mUKAs were performed on patients with primary KOA of the medial compartment who, after a 3-month conservative treatment including physical therapy, intra-articular cortisone injections, rest, and anti-inflammatory medications, still experienced substantial pain. After a review of their medical history, a physical examination, and a preoperative radiographic assessment, these patients were considered eligible for mUKA [36].

### Inclusion and exclusion criteria

We enrolled all the patients with a 5-year follow-up who met the classic mUKA selection criteria described by Kozinn and Scott in 1989 [37], i.e., a preoperative mechanical axis deformity of less than 10° in varus or 5° in valgus and a flexion contracture of less than 15°. Further criteria were an intact/competent anterior cruciate ligament, an intact lateral compartment, patellofemoral changes of no greater than grade II or III according to the Albach classification [38], and trochlear wear up to grade IV, provided it presented a central distribution [39].

Patients with primary lateral KOA, a history of complex knee surgery, significant trauma, inflammatory arthropathy, ataxia or neurological disease, psychiatric disorders (or who were in treatment), symptomatic KOA in the contralateral knee, and those who had required bilateral UKA or mUKA revision for aseptic loosening or infection during follow-up were excluded.

All patients provided their signed informed consent.

### Surgical procedure

All patients received the Link Sled® fixed-bearing prosthesis (LINK, Hamburg, Germany) with an MB or an AP cemented tibial component. All procedures were performed under spinal anesthesia associated with an adductor canal nerve block. A tourniquet was used in all cases. Surgeries were performed through an 8- to 10-cm limited medial midvastus approach without lateral patellar subluxation using a Link Mitus® ART (anatomic reconstruction technique) instrument set for minimally invasive surgery. The tibial cut was performed first to remove 5 mm of thickness from the most degraded portion of the plateau. The sagittal inclination was set according to the native tibial slope. Femoral preparation was performed by removing the cartilage layer with a saw blade. The femoral component was oriented according to the condyle anatomy. The aim of the procedure was to achieve an equal flexion–extension gap and to restore normal leg alignment. All implants were cemented. A suction drain was placed in all patients and removed the day after surgery.

### Rehabilitation protocol

Patients initiated rehabilitation on the first postoperative day. They engaged in a daily 90-min session with a physiotherapist in the morning and in an additional 90-min session without supervision in the afternoon. Immediate weight-bearing was prescribed. Crutches were indicated for the first month. Upon discharge, typically between day 3 or 4. The discharge was mutually agreed upon by the orthopedist, physiotherapist, internal medicine specialist, and patient, taking into consideration the clinical condition and attainment of short-term rehabilitation goals.

### Clinical outcomes

Key preoperative and postoperative variables were collected by qualified personnel of the Orthopedic Department. Patients were assessed preoperatively and then at 12 months and 5 years with five major patient-reported outcome measures (PROMs): the Knee Society Function Score (KSFS), the Knee Society Knee Score (KSKS) [40], and the Oxford Knee Society (OKS) Score [41, 42] as well as the physical component summary (PCS) and mental component summary (MCS) of the Short Form 36 Health Survey (SF-36), which measures HRQL [43].

At 5 years, we assessed the Forgotten Joint Score-12 (FJS-12) [44], patient satisfaction (ranging from 1, not satisfied, to 10, completely satisfied), the presence of tibial pain (particularly under the prosthetic tibial plateau) on the Numerical Rating Scale (NRS), and static postural sway, mobility, and gait symmetry, the latter three measures using the Riablo^TM^ system.

### Static postural sway and gait symmetry

The Riablo^TM^ device is an adaptive system comprising multiple IMUs and a force platform, all wirelessly connected to a computer. It has been developed to enhance conventional rehabilitation programs by directing users while performing prescribed physical exercises through a video interface. Each IMU weighs 20 g. IMUs operate on the wireless Bluetooth™ communication protocol and have a sampling frequency of 50 Hz [35]. The sensors are held in place with elastic bands. We employed three bands, which were attached (i) to the chest at the level of the mammillary line, and (ii) at the mid-thigh, and (iii) at mid-tibial level on the affected or the healthy limb, depending on the exercise. Data collected from five distinct exercises were then analyzed by the system to provide information on static postural sway, mobility, and gait symmetry:

1. The Timed Up & Go Test was used to assess the time the subject took to rise from a chair (seat height, 46 cm), walk a distance of 3 m, pivot, and return to a seated position in the chair [45].
2. The Sit-to-Stand Test, a practical test frequently employed as a measure of functional performance, was applied to assess the time required to move from a seated to a standing position a given number of times or to record the repetitions executed in a given time interval [46].
3. The Figure-of-8 Test was employed to evaluate walking proficiency in a single task: walking in a figure-of-8 pattern. Curved trajectories are key to navigating the figure-of-8 pattern, since the transition from a linear to a bending path and the adjustment of body movements in clockwise and counterclockwise directions replicates walking in everyday situations [47].
4. The Half-Turn Test has been incorporated into evaluations of mobility and balance for older individuals [48]. We used it to assess the ability to execute a swift and efficient turn. Participants were asked to take a few steps and then rapidly turn about to face the opposite direction. We measured the number of steps required to complete the 180° turn.
5. The Alternate Step Test, a version of the stool stepping task and one of the tests of the Berg Balance Scale [48], assesses coordinated weight shifting and serves as an indicator of lateral stability. Patients were required to rapidly mount a step measuring 18 cm in height and 40 cm in depth alternately with the left and the right unshod foot. We measured the time taken to accomplish eight steps.

Postural sway was evaluated using the Riablo™ stabilometric platform, where two pressure matrices, each fitted with 160 pressure sensors, record weight in a gradual manner. The maximum weight the pressure board can bear is 200 kg between the matrices. The platform communicates with the software through a Bluetooth protocol.

### Statistical analysis

Data analysis was performed using Microsoft Excel (2019) in conjunction with the XLSTAT resource pack (XLSTAT Premium, Addinsoft, New York, NY, USA). A propensity-score-matching (PSM) analysis was employed to minimize variations in known covariates between the cohorts [49], since several retrospective arthroplasty studies have used this type of analysis to minimize selection bias [8, 27, 50–57]. The two patient groups were matched one-to-one by an optimal matching algorithm [58]. The algorithm identifies matched samples with the smallest average absolute distance across all matched pairs. This technique, regarded as an optimal method to evaluate differences between treatment groups, was applied to mitigate the impact of potential confounding variables [59]. Patients were considered suitable for matching if the propensity score discrepancy between the groups fell within the caliper radius of 0.01 × sigma. The strength of the association and 95% confidence intervals were determined. The variables on which the two groups were harmonized included gender and ASA class (categorical data), age, BMI, preoperative KSFSs, KSKSs, and the OKS, PCS, and MCS scores (quantitative data).

The Shapiro–Wilk test was applied to determine whether the data had a normal distribution. Calculated mean values and standardized mean differences (SMDs) were also obtained for all continuous data. A non-parametric test, the Mann–Whitney test for unpaired data, and the Wilcoxon signed-rank test for paired data were employed to evaluate significant differences in continuous variables between the groups. Categorical data were analyzed using the chi-square test. Discrepancies between the MB and AP groups were assessed by comparing the SMD before and after matching. A group was regarded as imbalanced for a particular covariate if the SMD exceeded 0.2 [49]. A significance level of *p* < 0.05 was considered statistically significant.

## Results

The search of the institutional database yielded 653 mUKAs with a 5-year follow-up; of these, 342 had used an MB component (MB-UKAs) and 311 an AP component (AP-UKAs). A total number of 136 MB-UKAs and 124 AP-UKAs met the study criteria. PSM analysis successfully matched 77 pairs of patients for gender, age, BMI, ASA class and preoperative KSFSs, KSKSs, and OKS, PCS, and MCS scores.

### Demographics

Before PSM analysis, the two groups (136 MB-UKA and 124 AP-UKA patients) showed an imbalance in terms of BMI (the SMD was 0.82) and age, which was significantly different (*p* = 0.010). After PSM analysis, where patients were matched 1:1, we had two similar groups devoid of significantly different preoperative, perioperative, and postoperative features (Table 1).

**Table 1:** Comparison of preoperative, perioperative, and postoperative demographics of the medial UKA patients

Variable	Before propensity score matching				After propensity score matching			
	MB-UKA group	AP-UKA group	*P* value	SMD	MB-UKA group	AP-UKA group	*P* value	SMD
Age, mean (SD) [range]	67.6 (9.6) [44–89]	64.6 (10.1) [40–89]	0.010	0.01	66.1 (9.1) [49–88]	67.1 (9.7) [48–89]	0.689	0.11
Gender								
Male (%)	61 (44.9)	48 (38.7)	0.316	0.04	32 (41.6)	30 (39.0)	0.742	0.05
Female (%)	75 (55.2)	76 (61.3)			45 (58.4)	47 (61.0)		
BMI (kg/m2), mean (SD) [range]	27.5 (4.5) [18.3–44.8]	27.7 (4.5) [20.1–39.3]	0.857	0.82	27.5 (5.1) [18.3–44.8]	27.8 (4.3) [20.1–39.3]	0.682	0.07
ASA class (%)								
ASA 1	20 (14.7)	20 (16.1)		0.04	11 (14.3)	10 (13.0)		0.04
ASA 2	102 (75.0)	90 (72.6)	0.906	0.06	58 (75.3)	55 (71.4)	0.629	0.09
ASA 3	14 (10.3)	14 (11.3)		0.03	8 (10.4)	12 (15.6)		0.16

*UKA* unicompartmental knee arthroplasty, *MB-UKA* patients implanted with the metal-backed component, *AP-UKA* patients implanted with the all-polyethylene component, *SD* standard deviation, *BMI* body mass index, *ASA* American Society of Anesthesiology, *SMD* standardized mean difference

### Clinical outcomes

Before PSM analysis, the two groups were imbalanced in terms of KSFSs, with an SMD of 0.30. After PSM analysis, there were no residual significant differences. The preoperative KSFSs, KSKSs, and OKS, PCS, and MCS scores were not significantly different either before or after matching (Table 2).

**Table 2:** Preoperative and follow-up clinical and functional data and outcome satisfaction of the medial UKA patients

Variable	Before propensity score matching				After propensity score matching			
	MB-UKA group	AP-UKA group	*P* value	SMD	MB-UKA group	AP-UKA group	*P* value	SMD
KSKS								
Preoperative, mean (SD) [range]	47.4 (16.5) [22–70]	45.9 (16.6) [21–71]	0.211	0.07	45.1 (16.1) [22–69]	45.6 (16.5) [21–71]	1	0.03
12 months, mean (SD) [range]	88.9 (9.9) [58–97]	87.9 (11.0) [56–97]	0.542		87.9 (10.8) [59–97]	88.0 (10.7) [59–97]	0.764	
5 years, mean (SD) [range]	90.4 (9.7) [62–100]	89.5 (9.5) [60–100]	0.027		89.5 (10.4) [62–100]	89.7 (9.4) [60–100]	0.873	
KSFS								
Preoperative, mean (SD) [range]	57.2 (16.3) [32–80]	55.7 (16.3) [31–81]	0.211	0.30	54.9 (15.9) [32–79]	55.6 (16.1) [31–81]	0.873	0.04
12 months, mean (SD) [range]	88.6 (10.1) [58–97]	87.4 (10.9) [56–97]	0.263		87.8 (10.8) [59–97]	87.6 (10.6) [59–97]	0.920	
5 years, mean (SD) [range]	90.8 (10.3) [59–100]	90.3 (11.4) [60–99]	0.089		90.7 (10.8) [59–99]	90.5 (11.2) [60–99]	0.881	
PCS								
Preoperative, mean (SD) [range]	36.1 (10.6) [10–53]	35.4 (10.6) [9–51]	0.435	0.11	36.8 (10.4) [10–53]	35.6 (10.6) [10–51]	0.624	0.12
12 months, mean (SD) [range]	51.4 (8.1) [24–64]	49.2 (8.9) [27–69]	0.003		51.8 (7.6) [29–62]	49.1 (9.2) [27–69]	0.032	
5 years, mean (SD) [range]	52.9 (8.3) [25–66]	48.6 (8.7) [24–59]	<0.001		52.4 (8.3) [26–66]	48.2 (8.3) [24–59]	<0.001	
MCS								
Preoperative, mean (SD) [range]	51.8 (9.6) [29–65]	50.7 (10.2) [28–65]	0.303	0.11	50.6 (10.2) [29–63]	51.0 (10.0) [29–64]	0.826	0.04
12 months, mean (SD) [range]	57.3 (9.5) [34–70]	56.2 (8.5) [26–65]	0.024		56.0 (10.2) [34–69]	56.6 (8.2) [36–65]	0.897	
5 years, mean (SD) [range]	55.5 (9.4) [32–71]	54.9 (8.5) [23–65]	0.332		54.2 (9.6) [32–68]	55.4 (8.0) [36–65]	0.447	
OKS								
Preoperative, mean (SD) [range]	32.9 (7.3) [17–49]	33.1 (7.9) [16–48]	0.728	0.03	32.7 (7.6) [18–47]	32.5 (8.3) [16–48]	0.992	0.03
12 months, mean (SD) [range]	21.7 (5.5) [10–36]	21.2 (5.6) [10–33]	0.968		21.5 (5.2) [11–35]	20.6 (5.5) [10–33]	0.384	
5 years, mean (SD) [range]	18.3 (4.5) [9–32]	17.4 (4.6) [8–27]	0.263		18.1 (4.3) [10–31]	17.1 (4.4) [8–27]	0.168	
FJS-12								
5 years, mean (SD) [range]	81.4 (18.7) [42–100]	72.6 (21.9) [32–100]	<0.001		82.9 (18.8) [42–100]	73.4 (22.5) [32–100]	0.015	
Tibial pain at 5 years								
Present (%)	12 (8.8)	48 (38.7)	<0.001		6 (7.8)	27 (35.1)	<0.001	
Absent (%)	124 (91.2)	76 (61.3)			71 (92.2)	50 (64.9)		
Satisfaction								
5 years, mean (SD) [range]	9.2 (0.8) [8–10]	8.2 (2.1) [4–10]	0.015		9.2 (0.8) [8–10]	8.3 (2.0) [4–10]	0.003	

*UKA* unicompartmental knee arthroplasty, *MB-UKA* patients implanted with the metal-backed component, *AP-UKA*: patients implanted with the all-polyethylene component; *SMD* standardized mean difference, *KSKS* Knee Society Knee Score, *SD* standard deviation, *KSFS* Knee Society Function Score, *PCS*: physical component summary, *MCS* mental component summary, *OKS* Oxford Knee Score, *FJS*-12 Forgotten Joint Score

After matching, the PCS score at 12 months was 51.8 ± 7.6 (range 29–62) in MB-UKA and 49.1 ± 9.2 (range 27–69) in AP-UKA patients (*p* = 0.032). At 5 years, it was 52.4 ± 8.3 (range 26–66) and 48.2 ± 8.3 (range 24–59), respectively (*p* < 0.001).

The FJS-12 score at 5 years was 82.9 ± 18.8 (range 42–100) in MB-UKA subjects and 73.4 ± 22.5 (range 32–100) in AP-UKA subjects (*p* = 0.015).

Tibial pain at 5 years was reported by 7.8% of MB-UKA and 35.1% of AP-UKA patients (*p* < 0.001).

Satisfaction at 5 years was 9.2 ± 0.8 (range 8–10) in MB-UKA patients and 8.3 ± 2.0 (range 4–10) in AP-UKA patients (*p* < 0.003).

### Static postural sway, mobility, and gait symmetry

Static postural sway at 5 years was 3.9 ± 2.1 cm (range 0.60–10.30) in the MB-UKA group and 5.4 ± 2.3 cm (range 1.1–10.5) in the AP-UKA group (*p* = 0.0002) (Table 3).

**Table 3:** Static postural sway, mobility, and gait symmetry in medial UKA patients at the 5-year follow-up

Variable	Before propensity score matching			After propensity score matching		
	MB-UKA group	AP-UKA group	*P* value	MB-UKA group	AP-UKA group	*P* value
Static postural sway (cm), mean (SD) [range]	4.0 (2.3) [0.6–10.3]	5.5 (2.4) [1.1–10.5]	< 0.001	3.9 (2.1) [0.6–10.3]	5.4 (2.3) [1.1–10.5]	0.0002
Mobility (%)	71.8 (18.8) [35–100]	73.4 (18.9) [35–100]	0.313	70.9 (18.6) [37–100]	74.4 (18.6) [35–100]	0.062
Gait symmetry (%)	92.9 (3.7) [86.2–100.0]	90.2 (5.3) [79.3–99.9]	< 0.001	92.7 (3.7) [86.2–100.0]	90.4 (5.4) [79.3–99.9]	0.006

*UKA* unicompartmental knee arthroplasty, MB-UKA patients implanted with the metal-backed component, *AP-UKA* patients implanted with the all-polyethylene component, *SD* standard deviation

Gait symmetry at 5 years was 92.7% ± 3.7 (range 86.2–100) in MB-UKA and 90.4% ± 5.4 (range 79.3–99.9) in AP-UKA patients (*p* = 0.006).

## Discussion

The main finding of this study was that patients who underwent UKA with an MB component reported better static postural sway and gait symmetry at 5 years than those who underwent UKA with an AP component.

At the same time point, the AP patients also reported a significantly higher prevalence of tibial pain; they had significantly worse FJS-12 and PCS scores and were significantly less satisfied with their outcome. There were no other significant differences in the functional measures analyzed.

To the best of our knowledge, this is the first study to use a motion analysis system to compare the functional outcomes of UKA patients implanted with different tibial components.

Novel motion measurement systems that are developed to provide an objective description and quantitative assessment of patient motor functions and abilities always require validation. Several IMU-based techniques have been devised to track lower-limb joint movement, but only a few have been compared to stereophotogrammetry, the gold standard. The sensitivity and accuracy of the Riablo™ system, used in this study, have been validated by Leardini and co-workers [35]. In particular, the authors compared knee and chest angular measurements with the corresponding gait measures. They demonstrated that the IMU-based Riablo™ system makes minimal errors when measuring joint rotations, even in self-worn conditions. The authors concluded that the system is suitable for use in routine lower-limb joint rehabilitation, in patients who have received orthopedic treatment, and in those recovering from injury. The device has also been used as an aid to improve conventional rehabilitation in patients with neurological conditions [60, 61].

The advantages of these devices include cost-effectiveness compared to gait analysis equipment, a compact size and light weight, and the elimination of constraints related to the testing environment, extending it beyond the confines of the laboratory [34].

Wearable inertial devices for human motion analysis find widespread application in several areas. These include gait analysis (which is further subdivided into upright gait stability or dynamic balance assessment, measurement of spatio-temporal variables of gait, and evaluation of lower limb joint kinematics during gait); stabilometry (focusing on static balance assessment); instrumented clinical tests; assessment of upper-body mobility; monitoring of daily life activities; and evaluation of tremors [33].

At 5 years, the groups showed significantly different FJS-12 scores. The higher scores of the MB-UKA patients were comparable to those published by Longo et al. [62]. The lower FJS-12 scores of the AP-UKA patients may be related to the higher incidence of tibial pain in this group, possibly as a consequence of the greater proximal tibial strain, which has also been demonstrated in those patients compared to MB-UKA patients in a biomechanical study [63].

The finding that well-aligned AP-UKA implants involved greater bone deformation than misaligned MB-UKA implants [22] suggests that greater tibial bone strain and microdamage may contribute to component loosening [64] or unexplained pain [23], which may be the cause of the lower satisfaction and FJS-12 and PCS scores of our AP group. Clearly, pain could also affect the gait patterns and the ability to stand upright for a prolonged period, as shown by Riablo™ analysis.

Several studies have measured the postoperative functional scores of MB-UKA and AP-UKA patients.

Koh and colleagues found no significant differences in clinical and radiological outcomes at 2 years, including the Knee Society Score (KSS) and the Western Ontario McMaster University Osteoarthritis Index (WOMAC) scores [14].

In addition, a randomized study by Hutt et al. reported no differences in the Knee Osteoarthritis Outcome Score (KOOS) at a mean follow-up of 6.4 years [16].

We also failed to find significant differences in the KSKSs, the KSFSs, and the OKS and MCS scores, of which the latter measures HRQL (SF-36 score).

This finding contrasts with a retrospective study by Scott and co-workers, who reported no significant differences in SF-12 and PCS scores between 173 MB and 82 AP patients at 5 years [24]. Another study found better KSSs and KOOSs and lower pain in MB-UKA than in AP-UKA patients at a follow-up of more than 10 years as well as no significant differences in knee range of motion [65]. Lee et al., employing PSM, did not report any statistically significant differences between MB-UKA and AP-UKA patients in terms of outcomes, quality of life, or 10-year implant survival [27].

The main strength of our study is that all procedures were performed by highly experienced surgeons working at a specialized, high-volume knee prosthetic surgery center. All patients followed identical preoperative protocols, underwent the same implantation procedure, and followed a standardized rehabilitation protocol. The meticulous application of stringent inclusion and exclusion criteria, ensuring a highly homogeneous patient cohort, and the application of PSM, which—though yielding a limited number of patient pairs—effectively minimized confounding factors, are additional strengths.

The study’s limitations include, first of all, its retrospective design, since PSM minimized but clearly could not rule out selection bias. Patient number and follow-up duration are also limited.

Our findings demonstrate by movement analysis that MB-UKA provides greater stability and gait symmetry, even though both tibial components ensure good functional outcomes. Further studies are needed to establish which component provides better clinical results in the short, medium, and long term.

## Conclusions

At 5-year follow-up the static sway and gait symmetry demonstrated significant differences in between fixed-bearing AP and MB tibial components. Despite nearly overlapping functional outcomes, the MB-UKA group suffered less tibial pain, had better FJS-12 and PCS scores, and was more satisfied than the AP-UKA group.

## Data Availability

The datasets used and/or analyzed during the current study are available from the corresponding author on reasonable request.

## Abbreviations

mUKA: Medial unicompartmental knee arthroplasty
MB: Metal-backed
AP: All-polyethylene
PROMs: Patient-reported outcome measures
PSM: Propensity score matching
PCS: Physical component summary
FJS-12: Forgotten Joint Score-12
NRS: Numeric Rating Scale
KOA: Osteoarthritis of the knee
UKA: Unicompartmental knee arthroplasty
TKA: Total knee arthroplasty
IMU: Inertial measurement unit
HRQL: Health-related quality of life
BMI: Body mass index
ASA: American Society of Anesthesiologists
KSFS: Knee Society Function Score
KSKS: Knee Society Knee Score
OKS: Oxford Knee Society
MCS: Mental component summary
SF: Short Form
SMD: Standardized mean difference
SD: Standard deviation
KSS: Knee Society Score
WOMAC: Western Ontario McMaster University Osteoarthritis Index
KOOS: Knee Osteoarthritis Outcome Score
